# An Effectiveness Trial of STEADI in an Outpatient, Primary Care Practice

**DOI:** 10.1093/geroni/igaa057.2799

**Published:** 2020-12-16

**Authors:** David Rein, Madeleine Hackney, Michele Dougherty, Camille Vaughan, Laurie Imhof, Theodore Johnson, The Emory Clinic at St. Josephs STEADI Implementation Group

**Affiliations:** 1 NORC, Atlanta, Georgia, United States; 2 Emory University, Atlanta, Georgia, United States; 3 NORC at the University of Chicago, Bethesda, Georgia, United States; 4 NORC at the University of Chicago, Chicago, Illinois, United States; 5 Emory University School of Medicine, Atlanta, Georgia, United States; 6 Emory at Saint Joseph’s-Primary Care, Atlanta, Georgia, United States

## Abstract

The STEADI Options trial uses a randomized, controlled-trial design to assess the effectiveness and cost-effectiveness of the STEADI Initiative . Beginning March, 2020, we will randomize 3,000 adults ≥ 65 years of age at risk for falls seen in an Emory Clinic primary care practice to: (1) full STEADI; (2) a STEADI-derived gait, balance, and strength assessment with physical therapy referrals; (3) a STEADI-derived medication review and management; or (4) usual care. This presentation will discuss decisions made by the study team to facilitate implementation of STEADI including electronically conducting screening prior to the date of encounter, the use of dedicated nursing staff to conduct assessments, implementation of strength, balance, orthostatic hypotension, and vision testing, methods to facilitate medication review, and communication of assessment information to providers. The results from this study will be used to estimate the impact of STEADI on falls, service utilization, and costs over one year.

